# Case Report: Bicondylar conjoined Hoffa fracture with incarcerated patella

**DOI:** 10.3389/fsurg.2025.1480070

**Published:** 2025-02-13

**Authors:** Wissem Mansouri, Jean Darnaudet, Romain Huguet, Alban Fouasson-Chailloux, Vincent Crenn

**Affiliations:** ^1^ Department of Orthopedic and Traumatological Clinic, Nantes University, Nantes University Hospital (CHU Nantes), Nantes, France; ^2^ Department of Physical Medicine and Rehabilitation, Nantes University, Nantes University Hospital (CHU Nantes), Nantes, France; ^3^ CRCI2NA (Center for Cancer Research and Immunology Nantes-Angers), INSERM, UMR 1307, CNRS UMR 6075-Team 9 CHILD (CHromatin and Transcriptional Deregulation in Pediatric Bone Sarcoma), Nantes University, Nantes, France

**Keywords:** case report, Hoffa fracture, orthopaedic surgery, patellar fracture, traumatology

## Abstract

**Case:**

Hoffa fractures, uncommon injuries of the femoral condyle, sometimes involve both condyles, forming bicondylar fractures, typically from high-velocity trauma. We describe a 17-year-old male with an open conjoined bicondylar Hoffa fracture and a patellar fracture with incarceration following a road traffic accident. Emergency treatments included debridement, irrigation, and cannulated screw fixation. Postoperative care involved controlled range-of-motion exercises and specialized rehabilitation.

**Conclusion:**

Despite its rare and severe nature, this conjoined bicondylar Hoffa fracture with patellar incarceration was successfully managed, showing excellent recovery. CT scans are vital for accurate injury definition and surgical planning. Anatomical reduction and rigid fixation enable early mobilization and excellent long-term outcomes.

## Introduction

Hoffa fracture is a rare coronal-plane fracture of the femoral condyle that was first described by Hoffa in 1904 (1). This fracture can involve both the medial and lateral condyles and present as a conjoined bicondylar Hoffa fracture. This type of fracture is typically associated with high-velocity trauma (2). The proposed mechanism involves axially directed shear forces in a flexed knee, often accompanied by trauma to the extensor mechanism (3). We report a case of open conjoined bicondylar Hoffa fracture associated with fracture and incarceration of the patella. While similar cases exist in the literature, none exactly matches this traumatic injury. This report aimed to discuss the management, clinical presentation, and prognostic outcomes of this unique diagnostic entity. Written informed consent was obtained from the patients and their parents for publication of this case report.

## Case description

**Figure 1:**
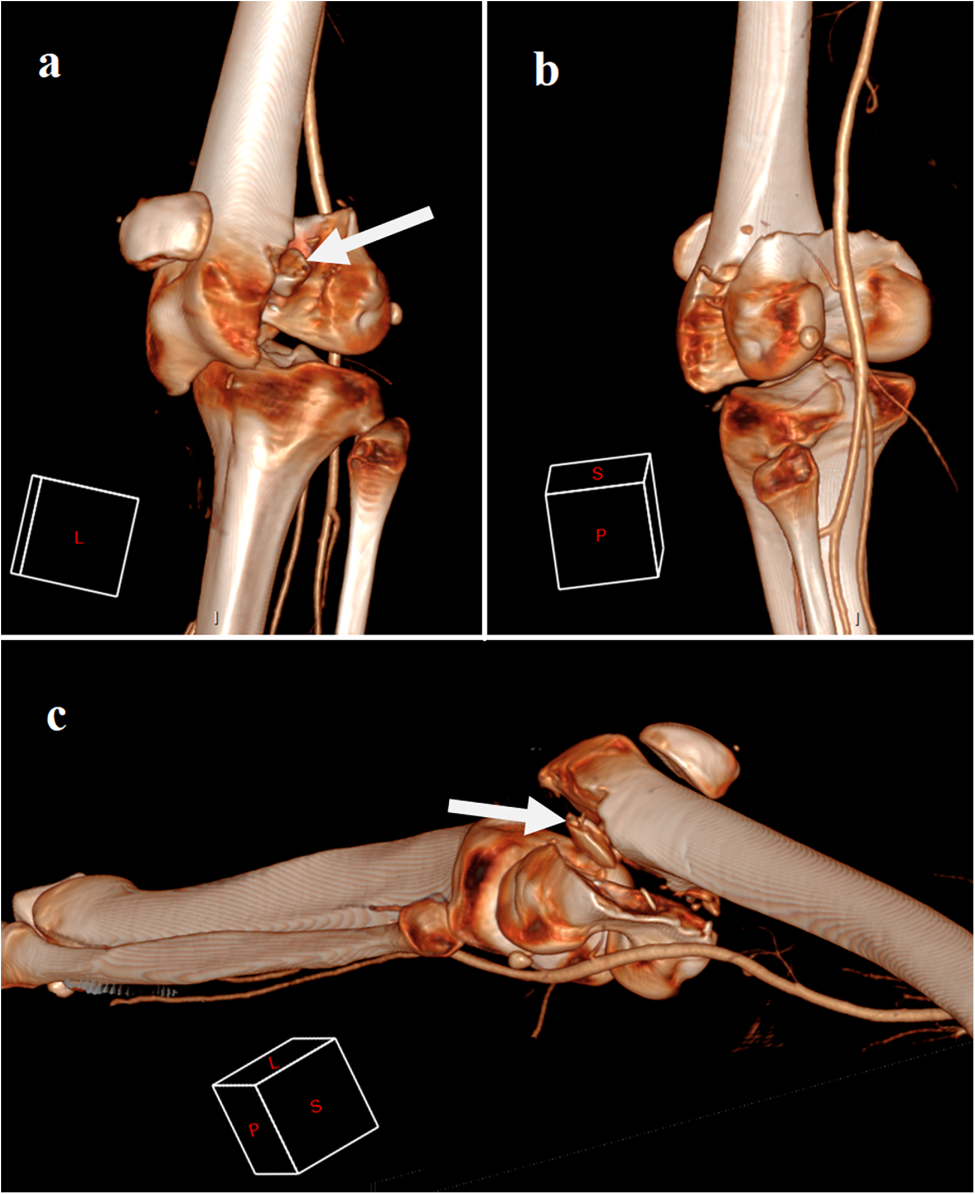
Ct scan 3D reconstruction showing conjoined bicondylar fracture with transverse patellar fracture and incarceration, in lateral view **(a)**, posterior view **(b)**, and supero-postero-lateral view **(c)** white arrows showing the patellar distal fragment incarceration.

**Figure 2:**
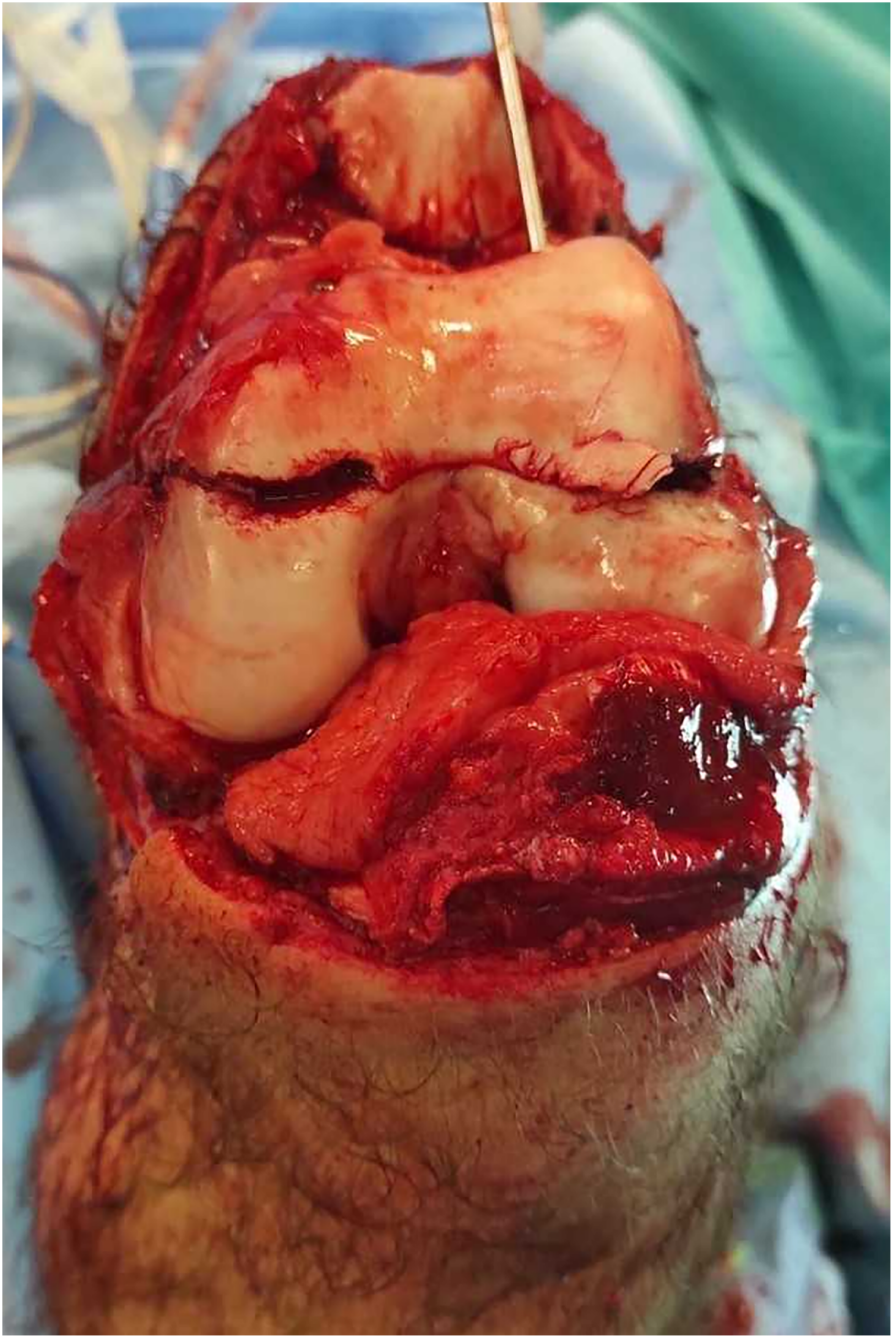
Open view through the transverse patellar fracture approach after complete debridement, irrigation and stabilisation with cannulated screws of the conjoined bicondylar fracture, showing minimal osteochondral defect at the trochleo-condylar junction.

A 17-year-old patient was involved in a road traffic accident while being seated as a front passenger in a vehicle. The patient arrived at the hospital and was already intubated and admitted to the intensive care unit. Physical examination and whole-body CT tomography revealed a Gustilo grade IIIA (4) open coronal plane fracture of the distal femur with an intact bridge of bone, bicondylar Hoffa fracture in the left knee, associated with a transverse patella fracture and incarceration of the distal fragment (Figure 1). The patient also had closed fractures of the contralateral tibia and fibula. The patient underwent emergency surgery within four hours post-trauma. Thorough debridement and extensive irrigation of the knee joint were then performed. Joint exploration revealed no meniscal or central pivot lesions. The coexistence of patellar fractures facilitated the surgical approach to both condyles (Figure 2).

The initial step in the surgical management involved the deimpaction of the distal patellar fragment from the fracture site. This was achieved by maintaining full knee extension and applying posterior-anterior pressure to the distal femur, which facilitated the opening of the fracture site and allowed for the successful deimpaction of the patella. Following this, knee flexion was employed to reduce the fracture. Two Müller forceps were placed on either side of the fracture to assist in reduction.

Fracture stabilization was initially performed using K-wires, which were then utilized as guides for subsequent osteosynthesis with 6.5 mm cannulated screws. Three screws were inserted along an antero-posterior trajectory. The first screw was positioned in the lateral condyle of the femur, while the second screw was inserted lower relative to the first, in alignment with the oblique fracture line, and aimed at the medial condyle. A third screw was placed between the first two to ensure optimal stability of the osteosynthesis construct.

**Figure 3:**
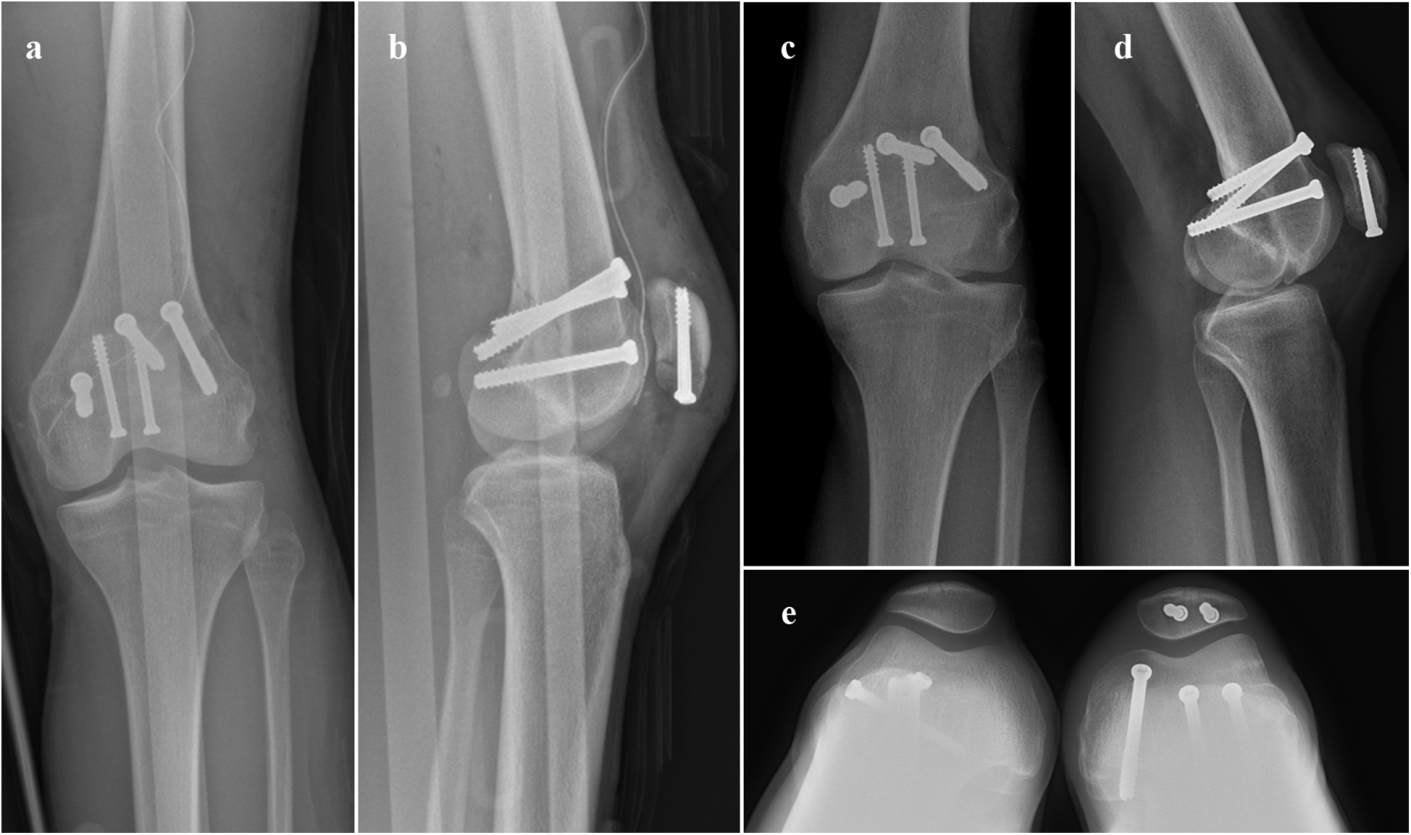
Immediate postoperative x-rays examination AP **(a)** and lateral view **(b)**; and postoperative x-rays at 6 months follow-up AP **(c)**, lateral **(d)** and patellofemoral view **(e)**.

An antero-posterior trajectory was chosen for screw placement, as it provided the most perpendicular alignment to the oblique fracture line, which was not perfectly vertical, as typically seen in a Letenneur type I fracture. This approach also helped preserve the integrity of the articular cartilage, which is crucial given its role in weight-bearing. In addition, intraosseous cannulated screws were selected for patellar fixation over cerclage wiring, to minimize hardware exposure, especially considering the initially open nature of the fracture (Figure 3).

Additionally, contralateral leg fracture was managed by upward pinning of the fibula and placement of a locked intramedullary tibial nail. Antibiotic prophylaxis with intravenous amoxicillin and clavulanic acid was administered for five days.

After surgery, the knee was immobilized with an extension splint, but immediate controlled range-of-motion exercises were initiated in the first week. The patient was discharged from a specialized rehabilitation center. Mobilization of the knee was allowed, with flexion limited to 60°, for the first three weeks. Six weeks post-surgery, the patient achieved 130° of active flexion and was pain-free, enabling progressive resumption of weight-bearing activities.

**Figure 4:**
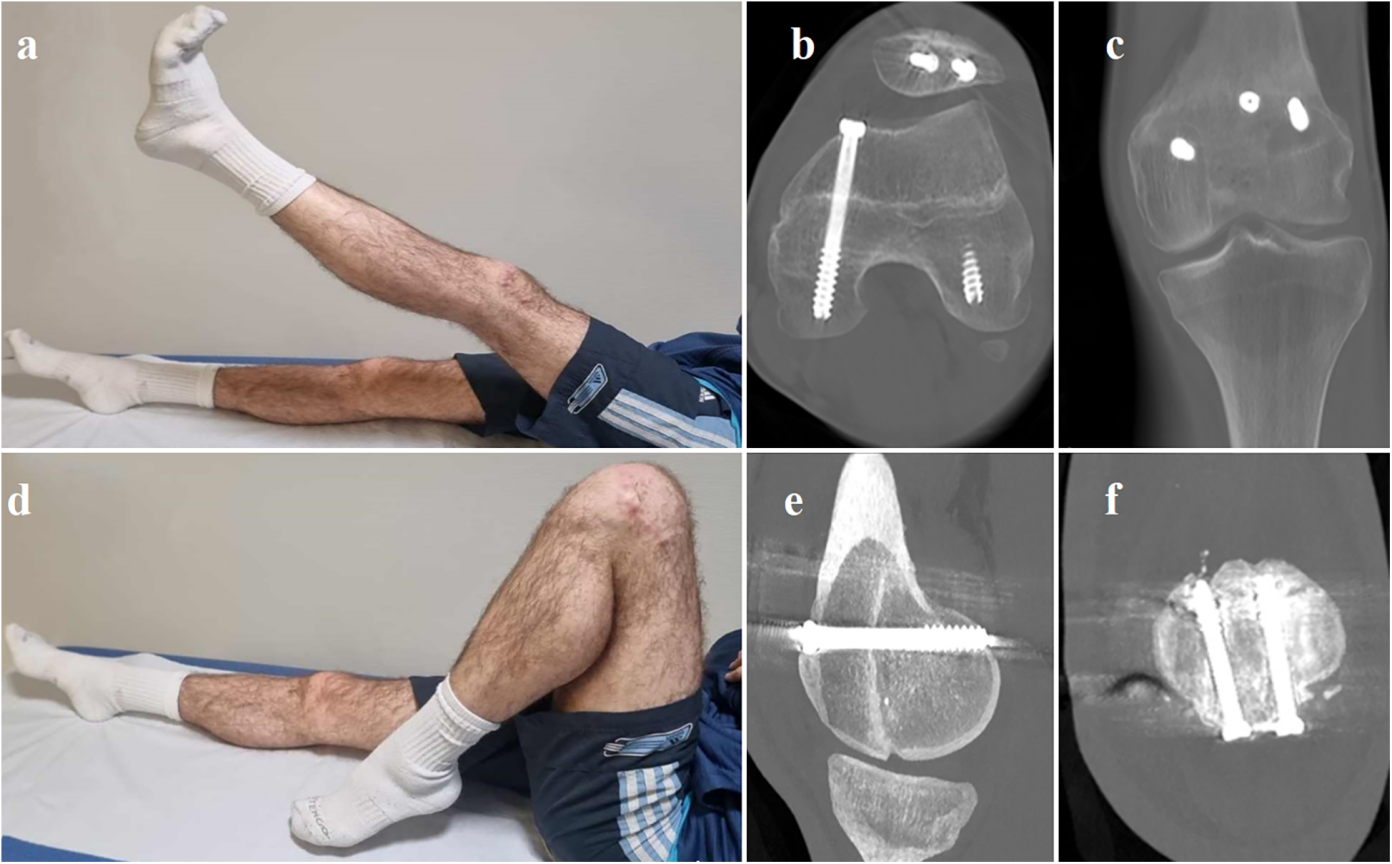
Clinical photographs showing the patient performing a full range of active motion in a supine position to assess knee function and muscle strength at the 6-month follow-up **(a** and **d)**. Axial **(b)**, coronal **(c)**, and sagittal **(e)** CT scan views at the 6-month follow-up illustrate the position of the implants and postoperative bone consolidation. Coronal view of the healed patellar fracture at the 6-month follow-up **(f)**.

Six months after the procedure, functional and radiographic results were excellent, with a range of motion from 0° to 130°. The patient demonstrated excellent outcomes based on the KOOS (Knee Injury and Osteoarthritis Outcome Score) evaluation (5). Scores on each subscale were notably high, with a pain score of 95, indicating minimal discomfort. The symptoms were minimal, reflected by a symptom score of 90. The patient exhibited exceptional functionality in daily activities, achieving a score of 95 in Activities of Daily Living (ADL). While the patient resumed sports at a minimal level, their participation in recreational activities was robust, with an Sport/Rec score of 90. Finally, the patient reported a high Quality of Life related to knee health with a quality of life (QOL) score of 85. Radiography and CT tomography confirmed complete consolidation of the fracture (Figure 4).

## Discussion

Bicondylar Hoffa fractures are particularly rare, with only a few cases reported in the literature, none of which involve open injuries with incarceration of the patella within the condylar fracture. Hoffa fractures are classified as type B3 within the AO/ASIF system (6). There is no classification in the literature describing bilateral conjoined or non-conjoined Hoffa fractures.

This distinctive fracture configuration is the result of direct anteroposterior forces applied to the flexed knee. The pattern of the condylar fracture depends on the degree of knee flexion at the time of impact. In Hoffa fractures, owing to physiological genu valgum, the lateral femoral condyle is predisposed to direct shearing forces, increasing the likelihood of fracture. When a flexed knee is subjected to a posterior and upward directed force without a varus or valgus component, it can lead to bicondylar Hoffa fracture. The degree of knee flexion directly correlated with the distance of the fracture line from the posterior femoral cortex. The extent of extensor mechanism injury is also determined by the degree of knee flexion at the moment of impact. Injuries can include rupture of the extensor mechanism, inferior patellar pole fractures, and even patellar dislocation (2, 3). Other associated injuries have also been reported, such as tibial spine fractures and damage to the ligamentous structures of the knee joint. In our case, the impact force was likely significant, causing transverse patellar fracture and incarceration of the distal patellar fragment within the femoral fracture site. This type of fracture usually originates from severe high-energy trauma secondary to motor vehicle collisions, work-related accidents, or falls (3, 7–9). They are often associated with other fractures, such as femoral and pelvic fractures, as a part of polytrauma. Therefore, a comprehensive management approach and hemodynamic stabilization are required. A CT scan demonstrated a bicondylar Hoffa fracture, with both fragments connected by a bridge of bone, forming the roof of the intercondylar notch. This helped us to understand the origin of the incarcerated bone within the femoral fracture. Using 3D reconstruction, a CT scan is the investigation of choice to accurately define the fracture pattern and comminution as well as to plan the surgical approach.

The treatment of displaced bicondylar Hoffa fractures is surgical. There is no place for non-operative treatment. Anatomical reduction of the articular surface, stable fixation, and early mobilization should be the treatment goals. Although there is no consensus, most authors recommend treating conjoined bicondylar Hoffa fractures by open reduction and internal fixation, particularly if the fractures are displaced by more than 3 mm (7–11). Some studies suggest that posterior-to-anterior directed screws may be more stable (12) in conventional lateral Hoffa fractures based on mechanical study experimentation. In our case, the patient presented with a Gustilo IIIA open fracture, which provided optimal exposure to both condyles through extensor mechanism rupture and patellar fracture. The method chosen was to fix the fracture in the anterior-to-posterior direction under direct vision and fluoroscopic guidance. Internal fixation with three antero-posterior cancellous lag screws seemed sufficient due to the high posterior fragment volume and its initial stability after reduction. This fixation allowed for immediate controlled motion. Hoffa fractures commonly present with other associated fractures (8), which suggests that the therapeutic outcome of the fracture is significantly influenced by the result of treating the accompanying injuries, as in our case with the patellar incarcerated fracture, which was fixed with two 5 mm diameter retrograde cannulated screws. Cannulated screws minimize the exposure of material to the primary skin aperture. As demonstrated by Carpenter et al. the mechanical strength of cannulated screw fixation is comparable to that of wire and cerclage fixation (13). Additionally, Hoshino et al. reported no difference in the complication rates between the two techniques, although the incidence of pain associated with the fixation material was twice as high in the pin and cerclage group (14).

In our case, early mobilization without weight-bearing yielded favorable results, mainly due to stable osteosynthesis and preservation of the patellofemoral ligaments (15). This approach facilitated repair of the knee extensor apparatus, enabling the patient to achieve full range of motion. Six months after surgery, the patient successfully returned to sport. Although there is no established consensus on the post-operative rehabilitation strategy for conjoined Hoffa bicondylar fractures, particularly when accompanied by an incarcerated patella fracture, several studies (2, 3, 16) have shown that correct stabilization enables early mobilization and contributes to better functional outcomes.

## Conclusion

We report a unique case of an open conjoined bicondylar Hoffa fracture with patellar fracture incarceration that was successfully managed with internal fixation with excellent outcomes. We believe that such fractures occur when a flexed knee is subjected to a posterior and upward-directed force without any varus or valgus components. CT is essential for accurately defining the injury and planning the surgical approach. Anatomical reduction and rigid internal fixation allow for early mobilization and excellent long-term results.

## Data Availability

The original contributions presented in the study are included in the article/Supplementary Material, further inquiries can be directed to the corresponding author.
